# Early exposure to maternal stress and risk for atopic dermatitis in children: A systematic review and meta‐analysis

**DOI:** 10.1002/clt2.12346

**Published:** 2024-03-15

**Authors:** Yuan Ai, Jichong Huang, Ting Ting Zhu

**Affiliations:** ^1^ Department of Pediatrics West China Second University Hospital Sichuan University Chengdu China; ^2^ Key Laboratory of Obstetric & Gynecologic and Pediatric Diseases and Birth Defects of Ministry of Education Sichuan University Chengdu Sichuan China

**Keywords:** atopic dermatitis, maternal, meta‐analysis, stress, systematic review

## Abstract

**Background:**

The incidence of atopic dermatitis (AD) in children is increasing. Early exposure to stress factors may be associated with the AD development. This study aimed to summarize studies that reported an association between stress exposure and AD development in later life.

**Methods and findings:**

A comprehensive literature search was performed using online databases (PubMed, EMBASE, PsycINFO, and Web of Science) for articles published up to May 1, 2023. Eligible studies were screened and selected based on the inclusion criteria. We incorporated cohort or case‐control studies published in English which explored the relationship between stress experienced by parents or children and AD. The pooled odds ratio (OR) was calculated according to the type of stress using a random‐effects model. Twenty‐two studies were included. AD was related to maternal distress (OR 1.29, 95% Confidence Interval [CI]: 1.13–1.47), maternal anxiety (OR 1.31, 95% CI: 1.18–1.46), and negative life events (OR 2.00, 95% CI: 1.46–2.76). Maternal depression during pregnancy was associated with AD (OR 1.21, 95% CI: 1.09–1.33), whereas no significant association was found for postpartum depression. Research on stress experienced by paternal or children is scare.

**Conclusions:**

Early maternal stress may potentially elevate the risk of AD in their offspring. Importantly, rigorously designed studies are required to corroborate the link between maternal stress and AD in children. These studies should aim to gather insights about the impact of stress during specific trimesters of pregnancy, postnatal stress, and paternal stress, and to identify potential prevention strategies.

## 1 INTRODUCTION

Atopic dermatitis (AD), also known as atopic eczema, is an inflammatory disorder of the skin that results in chronic, recurrent, or persistent edema, itching, excoriation, swelling, redness, and irritation.^1^ According to a recent systematic review of the prevalence and incidence of AD, focusing on data from the 21st century, the overall point prevalence of AD symptoms in children ranges from 1.7% to 32.8% and the 1‐year prevalence in children based on a doctor's diagnosis of AD ranges from 0.96% to 22.6%.^2^ The latest available data (Phase Three of the ISAAC study) show that the prevalence of AD has continuously increased in young children and in low‐income countries.^3^ Multiple aspects, including environmental hazards, genetic factors, and immune dysfunction, are involved in AD pathogenesis.^4^ However, the etiological factors are still being explored and remain unknown. Early exposure to stressful life events may alter inflammatory responses, leading to adverse health outcomes.^5^, ^6^ Stress is normally induced by a stimulus of a stressor that may arise from external events or from an individual's perception of their situation as stressful. Observational cohort studies have shown that maternal anxiety, depression, or work stress during pregnancy may increase the risk of AD in their children.^7^, ^8^, ^9^ The underlying mechanisms that elucidate these observed associations encompass immune system alterations, genetic modulation, and impacts on the hypothalamic‐pituitary‐adrenal axis. All these mechanisms are based on speculation and not fully understood.

Notably, a meta‐analysis indicated no association between prenatal anxiety and childhood AD.^10^ However, this study did not consider other stressful factors, such as work stress, divorce/separation, and economic problems. These adverse life events may predispose to stress/distress. Brew et al. reported that early‐life bereavement experienced by women was associated with early‐onset offspring asthma.^6^ Emerging studies in both animals and humans have suggested the impact of parental life experiences on the health of offspring.^11^, ^12^ Furthermore, it remains unclear whether the timing of stress exposure before AD onset influences the development of AD. Recent studies have indicated that maternal stress during various trimesters and postpartum depression occurring at different times may variably increase the risk of AD development. ^13^, ^14^ Thus, elucidating the relationship between stress and AD is crucial for exploring the pathogenesis of AD, and improving maternal/parental mental health and stress that could reduce the risk of AD in childhood.

This systematic review and meta‐analysis included a wide spectrum of stressors before child AD onset. We summarize the current evidence exploring the risk of AD in children who are exposed to maternal/parental stress during pregnancy or after delivery.

## 2 METHODS

This study was conducted according to the Preferred Reporting Items for Systematic Reviews and Meta‐Analyses (PRISMA) guidelines. The inclusion and exclusion criteria were based on PICO principles.

### 2.1 Eligibility criteria

We selected observational (cohort or case‐control) studies that were published in English, and investigated the association between stress experienced by parents (maternal or paternal) or children before AD onset (including pregnancy and after delivery) and AD. Participants were children below the age of 18 years and their parents. Stress included perceived stress (work stress, social stress, daily hassles, and negative life events) and adverse emotions (distress, anxiety, and depression). Stress can be objectively assessed using various scales or can be subjectively reported. When multiple studies overlapped in terms of study population and definitions of exposure and outcomes, we selected the study with the largest sample size. Studies in the formats of reviews, case reports, case‐series, and cross‐sectional studies, as well as abstracts, were excluded.

### 2.2 Literature search

Online databases, including PubMed, EMBASE, PsycINFO, and Web of Science, were searched for related studies published up to May 1, 2023. We used the following general terms: (maternal, prenatal, paternal, pregnancy) AND (stress, anxiety, depression, stressful events, psychology) AND (eczema, atopic dermatitis). The details of the search strategies are provided in the Supporting Information S2. Two reviewers (J. H. and Y. A.) independently screened titles and abstracts, and the selection of any potential studies was confirmed by full‐text reading. Any discrepancies were resolved through discussion with a third author (T. Z.).

### 2.3 Data extraction and quality assessment

Data extraction and quality assessments were conducted by two reviewers, J. H. and T. Z. The study characteristics extracted included the first author, year of publication, country, study design, and sample size, along with exposure parameters (type of stress, time of stress, and stress measurement) and outcome parameters (age at AD diagnosis). Both crude and adjusted odds ratios (aOR), as well as the corresponding 95% confidence intervals (CI) from each study, were also extracted. In cases where studies calculated multiple adjusted models, the aOR from the model employing the most covariates was reported. Furthermore, if studies investigated multiple exposures and presented various results, the OR for the most severe exposure was reported. Notably, cohort and case‐control studies were assessed using the Newcastle–Ottawa scale.^15^ Quality assessment was independently performed by two reviewers (J. H. and Y. A.). Discrepancies in assessments were resolved by discussion until a consensus was reached (T. Z.).

### 2.4 Data synthesis

Quantitative synthesis was performed according to the exposure type. If more than two studies reported the estimated effect of the same exposure, the results were pooled. We chose aORs for pooling unless the included studies provided only crude ORs. A random‐effects model was used to combine the results, and Cochran's *Q* test and *I* ^2^ index were used to calculate statistical heterogeneity. A sensitivity analysis was conducted by sequentially removing each study and recalculating the pooled ORs. Funnel plots were only used for the association between stress (stress, distress, and adverse life events) and AD, as in other pooled analyses with fewer than 10 studies. Data analyses were performed using Review Manager Software, version 5.3.

## 3 RESULTS

As shown in Supplementary Figure S1, the selection process was conducted following the Preferred Reporting Items for Systematic Reviews and Meta‐Analyses flow diagram, with non‐duplicated studies retrieved from the online database. After screening the title, abstract, and full‐text gradually, 18 studies were excluded for reasons, such as no eczema data, cross‐sectional design,^8^, ^16^, ^17^ overlapped population,^18^ and adult AD,^19^ leaving 22 studies in the final analysis.

### 3.1 Study characteristic

The characteristics of the included studies are summarized in Table 1 and sorted according to the timing of the stress exposure. Main results of the included studies are presented in the Supplementary table (Table S1). Most of these were cohort studies, comprising 20 prospective cohort studies,^7^, ^9^, ^13^, ^14^, ^20^, ^21^, ^22^, ^23^, ^24^, ^25^, ^26^, ^27^, ^28^, ^29^, ^30^, ^31^, ^32^, ^33^, ^34^, ^35^ one retrospective cohort study,^36^ and one case‐control study.^37^ The study population comprised individuals from various countries, including Europe, the United States, China, and Australia. More than 80% of the studies were published after 2013. Maternal stress during pregnancy was the most commonly assessed type of stress, followed by maternal anxiety, depression, and stressful life events after delivery. The age of AD outcomes varied between studies, mostly before 6 years of age. The assessment of stress was determined through either health care contact for depression or anxiety or by employing a self‐reported questionnaire. The questionnaires used included the Screening Scale of the Trier Inventory of Chronic Stress (SSCS‐TICS) and Hospital Anxiety and Depression Scale (HADS) among others. The methods for assessing AD differed across studies, ranging from physician diagnoses to paternal or self‐report questionnaires.

**TABLE 1:** Characteristics of included studies.

Study	Design, country, *n*	Exposure (age measured)	Outcome (age measured)	Effect measure: Estimate (95% CI)
Prepregnancy				
El‐Heis 2017^20^	Prosp. Cohort UK, 2956	Maternal stress and mood (preconception and at 6 months postpartum)	AD at 6–12 m	OR: Preconception perceived stress affecting health 1.21 (1.08–1.35); stress in daily living 1.16 (1.03–1.30)
During pregnancy				
Cheng 2015^21^	Prosp. Cohort USA, 1125	Maternal psychological state (26 weeks gest)	AD within 12 m	OR: EPDS (≥15) 1.13 (0.43–2.99)
				STAI state (≥41) 1.01 (0.56–1.81)
Shen 2020^22^	Prosp. Cohort China, 1638	Maternal psychological state (1st, 2nd, and 3rd trimesters)	AD at 6 m	OR: High stress in the 2nd trimester 1.56 (1.08–2.25)
				Increased stress from the 1st to the 2nd trimester 2.05 (1.33–3.15)
				Increased stress from the 1st to the 3rd trimester 1.92 (1.22–3.00)
Shi 2023^13^	Prosp. Cohort China, 3252	Maternal perceived stress, anxiety, and depression (12–16, 32–34 weeks gest)	AD at 2, 6, 12 m	Eczema at 2 months: High stress during early pregnancy 1.30 (1.01–1.67); late pregnancy 1.64 (1.14–2.36)
Larsen 2014^23^	Prosp. Cohort Danish, 32,104	Maternal psychosocial work environment (15 weeks gest)	AD at 7 years	OR: High strain 1.15 (1.02–1.31)
Wang 2013^9^	Prosp. Cohort China, 11,962	Maternal employment status during pregnancy	AD at 3 years	OR: Work during pregnancy 1.38 (1.25–1.53)
				Very high ⁄high work stress 1.34 (1.16–1.54)
Elbert 2017^24^	Prosp. Cohort Netherlands, 5205	Maternal and paternal psychiatric symptoms during pregnancy (2nd trimester)	AD at birth to 10 years	Maternal: Overall psychiatric 1.54 (1.05, 2.57); anxiety 1.35 (1.04, 1.76); depression 1.29 (1.02, 1.64)
Smejda 2018^25^	Prosp. Cohort. Poland, 370	Maternal stress during pregnancy (20–24 weeks gest)	AD at 12 m	Perceived stress scale 0.98 (0.93–1.04)
				Social Readjustment Rating scale 0.99 (0.92–1.06)
Senter 2021^26^	Retrosp. Cohort. USA, 426	Maternal stress life events during pregnancy (12 months before delivery)	AD at 4–6 years	Current eczema: 1.08 (0.89–1.31) location‐specific AD 1.09 (0.78–1.52) ever AD 0.97 (0.87, 1.09).
Wen 2011^27^	Prosp. Cohort China, 1264	Maternal mental status during pregnancy (3rd trimester)	AD at 2 years	High stress 2.3 (1.1–5.3)
				Median stress 1.1 (0.5–2.8)
Hartwig 2014^28^	Prosp. Cohort Australian, 1264	Maternal negative life events during pregnancy (18 and 18–34 weeks gest)	AD at 6 or 14 years	Eczema at 14 years: 18w 1.18 (0.54–2.60)
				18–34w 4.19 (1.97–8.89)
Chang 2016^29^	Prosp. Cohort Korean, 973 and 1531	Prenatal depression, anxiety, and distress (36 weeks gest, last 30 pregnancy days)	AD at 4–5 years	Prenatally depressed 1.31 (1.02–1.69)
				Prenatally anxious 1.41 (1.06–1.89)
				Prenatal distress 1.86 (1.06–3.26
Sausenthaler 2009^7^	Prosp. Cohort German, 3004	Stress‐related maternal factors during pregnancy	AD at 0–6 years	OR: 0–2 years 1.48 (0.95–2.30)
				0–6 years 1.13 (0.71–1.79)
Braig 2016^30^	Prosp. Cohort German, 787	Maternal stress, depression, and anxiety during pregnancy	AD at 2 years	Hospital anxiety and depression scale (≥8 points): 1.4 (1.0–2.0); trier inventory of chronic stress, screening Scale (upper vs. low quarter): 1.5 (1.0–2.3)
Hamann 2019^31^	Case‐control. Denmark, 8602 eczema cases	Parental psychiatric disease during pregnancy	AD at 5 years	Maternal depression 1.15 (1.00–1.33)
				Maternal anxiety 1.38 (1.12–1.71)
Puosi 2021^32^	Prosp. Cohort Finland, 1305	Maternal psychological distress (at weeks gest 14, 24, 34, and at child age 2 years)	AD at 2 years	EPDS (high and decreasing) 1.37 (0.71–2.64)
				SCL‐90 (high and decreasing) 1.79(0.96–3.26)
Letourneau 2017^33^	Prosp. Cohort Canada, 294	Prenatal and postnatal maternal psychological distress (pregnancy and again at 3 months postpartum)	AD at 18 m	Pregnancy specific anxiety 2.78 (1.04–7.39)
				Postnatal anxiety 1.16 (1.01–1.33)
				Maternal unresponsiveness 1.35 (1.05–1.73)
van der Leek 2020^14^	Prosp. Cohort Canada, 12,587	Prenatal and postnatal distress of mother	AD at 5 years	Prenatal distress 1.27 (1.11–1.46)
				Postnatal, recurrent 1.28 (1.11–1.48)
				Postnatal, late‐onset 1.19 (1.06–1.34)
Postnatal				
McKenzie 2020^34^	Prosp. cohort USA, 4898	Adverse childhood Experiences (5‐, 9‐, and 15‐year)	AD at 5‐, 9‐, and 15 years	OR: The number of adverse experiences at 5 years: ≥3, 2.10 (1.52–2.89) at 9 years: ≥3,1.48 (1.09–2.01)
Bockelbrink 2006^35^	Prosp. Cohort German, 3097	Parental stressful life events within the first 2 years	AD at 4 years	OR: Divorce/separation 1.86 (1.09–3.19)
Wang 2016^36^	Prosp. Cohort China, 18,024	Postpartum depression at 6 months after delivery	AD at 3 years	OR: Postpartum depression 1.42 (1.21–1.66); maternal high stress 1.08 (0.92–1.28)
Yoon 2018^37^	Prosp. Cohort Korean, 1620	Perinatal negative life event (during past 12 months and within 4 m after delivery)	AD at 6 years	Eczema symptoms during the last 12 months in female 2.15 (1.24–3.71); eczema treatment during the last 12 months in female 2.08 (1.15–3.79)

Abbreviations: AD, atopic dermatitis; EPDS, the Edinburgh Postnatal Depression Scale; m, months; OR, odds ratio; Prosp, prospectively; Retrosp, retrospectively; STAI, State‐Trait Anxiety Inventory; UK, United Kingdom; USA, United States of America; wks gest, weeks of gestation; y, years.

Among the 22 studies deemed eligible for inclusion, only two explored the relationship between paternal stress and AD. Elbert et al.^24^ found no significant association between paternal psychiatric symptoms (including overall, depressive, and anxiety symptoms) during pregnancy and at 36 months post‐delivery with AD. Conversely, Hamann et al.^37^ observed that paternal depression during pregnancy was associated with an increased risk of a child's AD diagnosis (OR 1.16, 95% CI: 1.06–1.26), though no significant associations were found for paternal anxiety or contacts for psychiatric care. Only one study has investigated adverse events experienced by children directly during childhood and later AD development and reported increased odds of AD before 9 years of age.^32^

### 3.2 Association between maternal stressful factors and children with AD

#### 3.2.1 Maternal stress/distress

**FIGURE 1:**
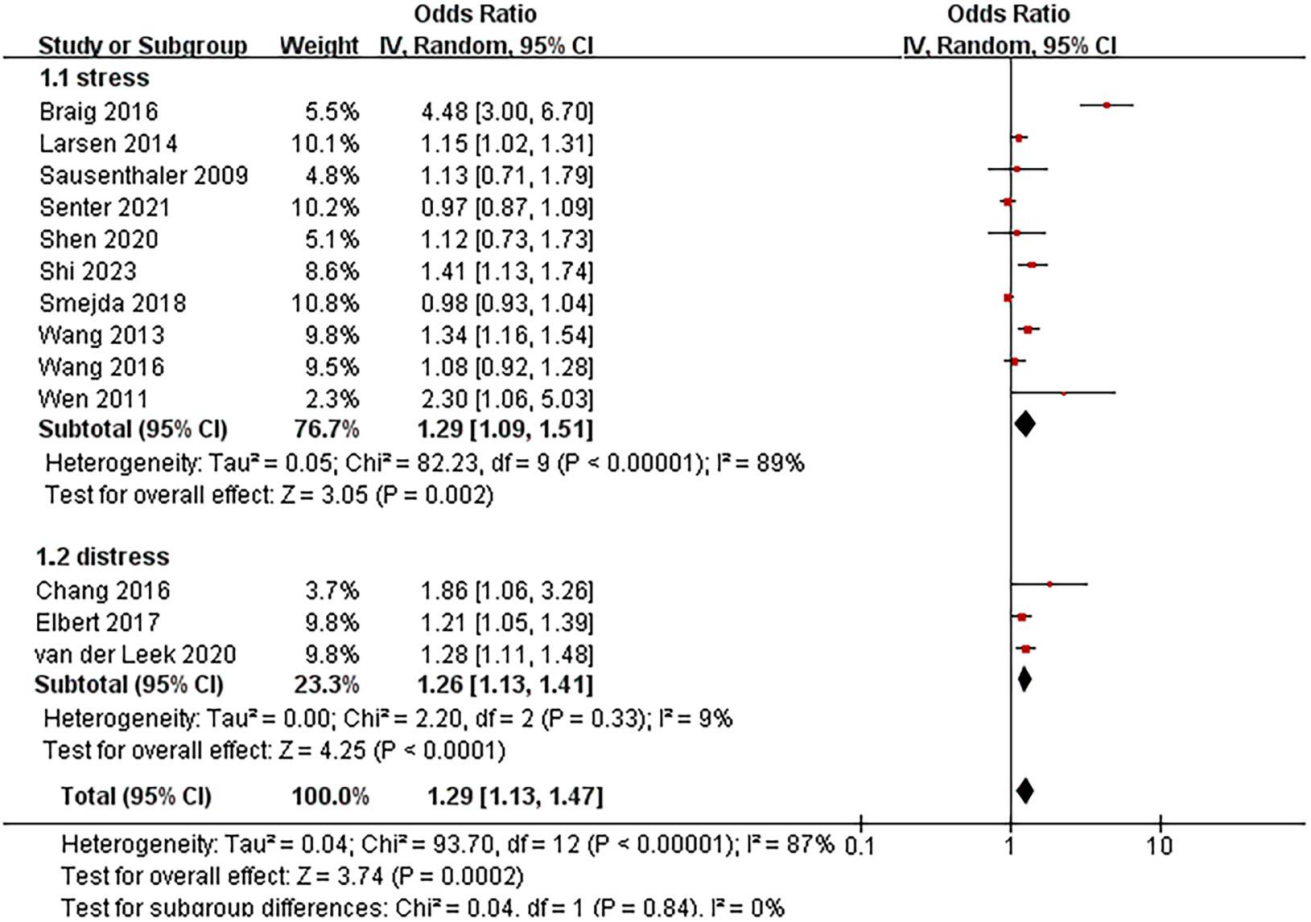
Forest plot for the association between maternal stress/distress and the development of atopic dermatitis in children.

Thirteen studies were included in this meta‐analysis (Figure 1). Infants born to mothers with stress during pregnancy and after delivery (OR: 1.29 95% CI: 1.09–1.51, *I* ^2^ = 89%) and distress during pregnancy (OR: 1.26; 95% CI: 1.13–1.41, *I* ^2^ = 9%) had a higher risk of AD compared to those who did not.

#### 3.2.2 Maternal anxiety

**FIGURE 2:**
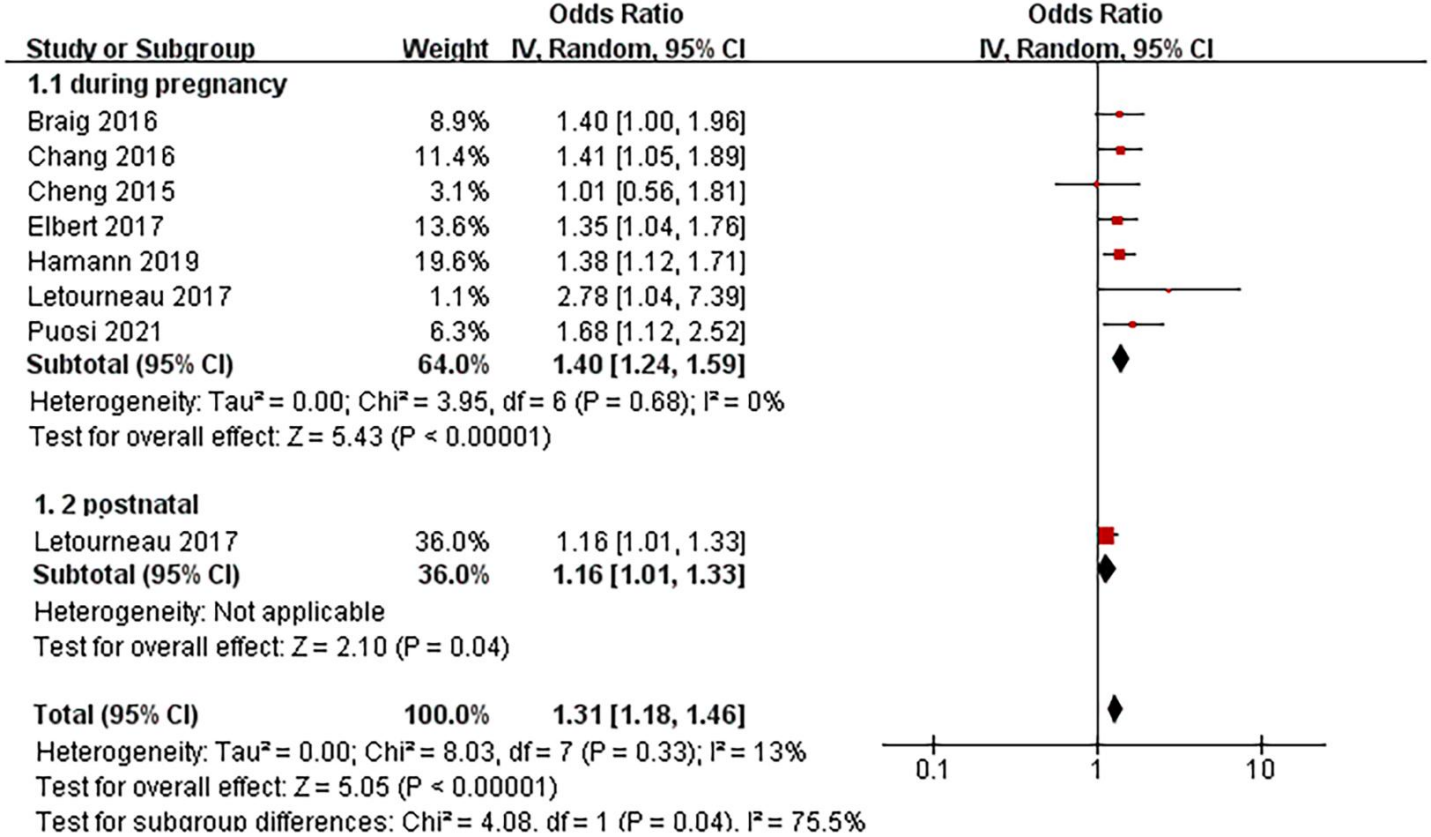
Forest plot for the association between maternal anxiety and the development of atopic dermatitis in children.

Eight studies assessed the association between maternal anxiety and AD, with one reporting that postnatal maternal anxiety increases the risk of AD (Figure 2). The pooled ORs of the remaining seven studies for the association between prenatal maternal anxiety during pregnancy and AD was 1.40 (95% CI: 1.24–1.59).

#### 3.2.3 Maternal depression

**FIGURE 3:**
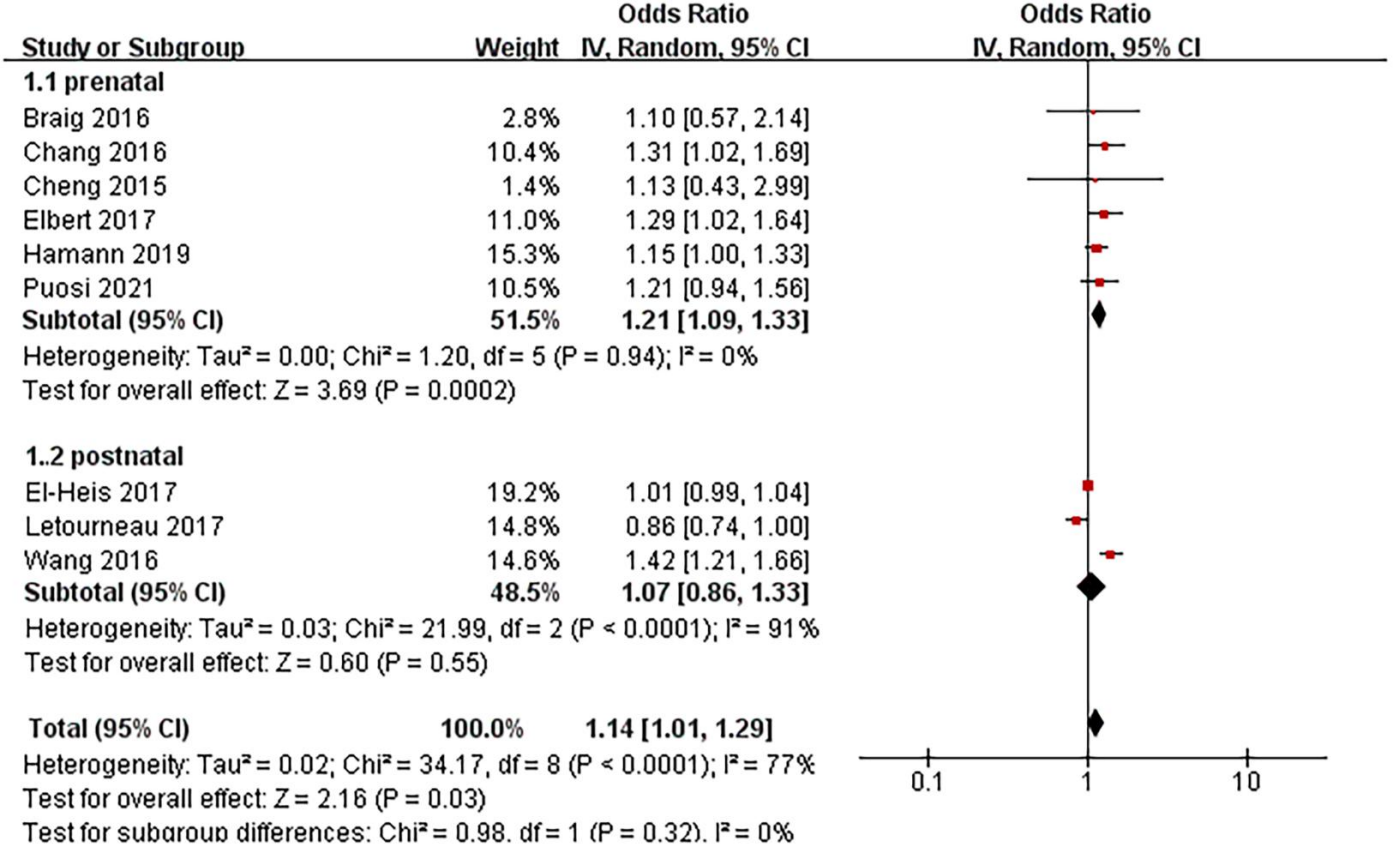
Forest plot for the association between maternal depression and the development of atopic dermatitis in children.

Six studies investigating prenatal depression and four examining postnatal depression were included in this meta‐analysis. Figure 3 displays the aggregated findings. Notably, only depression during pregnancy was significantly associated with an increased risk of AD in offspring (OR 1.21, 95% CI: 1.09–1.33; *I* ^2^ = 0%; *p* < 0.01). In contrast, no significant relationship was observed between postnatal depression and AD, and a considerable heterogeneity among the studies was evident (OR 1.07, 95% CI: 0.86–1.33; *I* ^2^ = 91%).

### 3.3 Adverse life event exposure

Adverse life events are defined as traumatic and stressful experiences encountered by a child or their mother, encompassing circumstances such as divorce, the death of a family member, job loss, emotional abuse, and economic hardship. Four studies were pooled for the meta‐estimate, and maternal exposure to adverse life events during pregnancy and after delivery was associated with an increased risk of AD in their offspring (OR 2.00, 95% CI: 1.46–2.76; *I* ^2^ = 46%) (Supplementary Figure S2).

### 3.4 Study quality

Overall, all included studies were graded as good, except for one study that was graded as fair^32^ (Supplementary Table S2). Methodological limitations included a lack of controls for additional confounders, such as maternal smoking and alcohol intake, and describing the response rate in cohort studies. Sensitivity analyses indicated that no individual study affected the combined results.

## 4 DISCUSSION

This is the first comprehensive systematic review summarizing 22 available studies that have investigated the association between stress exposure and AD in children. As the conclusions of these studies differed, a meta‐analysis was conducted to pool the study results. We found that children born to mothers with stress/mental health conditions during pregnancy had a higher risk of AD than those born to mothers without stress/mental diseases. Among the stress factors, adverse life events exposure resulted in the highest OR for AD development in offspring.

The exact mechanisms underlying the association between maternal stress and AD remain unknown; however, the core hypothesis is the relationship between stress and the immune system. Recently, a larger‐scale meta‐analysis across 12 datasets from 10 pregnancy cohorts revealed that prenatal maternal stressful life events are associated with cord blood differential methylation of CpGs in APTX, MyD88, and both UHRF1 and SDCCAG8 in the offspring, which are implicated in neurodegeneration and immune and cellular functions.^38^ Pregnancy has its unique biology and is a critical period for the biological transfer between mothers and infants.^39^ Elevated inflammatory and pro‐inflammatory biomarkers were observed when mothers experienced stress during the prenatal period.^40^, ^41^ AD is an inflammatory skin disease predominantly characterized by CD4+ T‐cell infiltrate in the lesional skin.^42^ AD is now recognized as a biphasic disease with Th2 cells predominating in the acute phase and a transition to Th1 cells characterizing the chronic phase.^43^ An animal study demonstrated that IL‐4 secretion was notably increased in the offspring of mice subjected to maternal stress during pregnancy. In these offsprings, Th2 cytokines generally prevailed, leading to an elevated Th2/Th1 ratio compared to offspring from mice not exposed to maternal stress during pregnancy.^44^ Exposure to stress, distress, and anxiety may lead to chronic activation of the maternal hypothalamic‐pituitary‐adrenal (HPA) axis, which then affects the fetal autonomic nervous system, HPA axis, and potentiation of a Th2 skewed immune milieu.^45^, ^46^ In addition, offspring born to mothers with anxiety, depression, or stress had different gut microbiome diversity and bifidobacterial abundance.^47^ Alteration in the gut microbiota may play a key role in the pathophysiological development of AD.^48^ Furthermore, paternal stress can impact the health of offspring through genetic inheritance, particularly by altering the sperm epigenome. This includes modifications in DNA methylation, histone modifications, sncRNAs, and sncRNAs‐associated alterations.^49^ Importantly, paternal stress might also indirectly affect the health outcomes of offspring by influencing maternal investment in their offspring.^50^ However, these mechanistic pathways are less well established in humans and should be explored further to establish the first instigators of immune activation in diverse maternal states.

Our search strategy and the meta‐analysis were consistent with those employed in other systematic studies investigating the impact of maternal psychosocial stress on the health of offspring. Notably, even in cases where children possessed a susceptibility gene for allergic diseases, only studies reporting outcomes of AD were included. The focus of a previous meta‐analysis was to explore the effects of maternal mental disorders (both anxiety and depression) on AD in offspring, and no significant association was found between them.^10^ In the present meta‐analysis, we focused on the impact of a wide spectrum of parental stress/distress in early life (during pregnancy and after delivery) on AD in offspring. Newly published cohort and case‐control studies were included, which enabled us to obtain more precise estimates. Few studies have examined the influence of paternal stress on children's stressful experiences during early life. As an important member of the family, paternal mental disorders may also result in alterations in maternal mood and lifestyle. Experiences of chronic and/or severe stress in early life, not only during pregnancy but also from neonatal to childhood, may increase the risk of poor health during late adolescence.^51^, ^52^

Cases of maternal stress identified in most primary studies were based on different tools. For example, adverse life events assessment in one study^37^ used a score by asking participants to select from 27 life events, including separation or divorce, death of a close relative or friend, and financial problems. In another study^35^ included only the death of a family member and divorce. Furthermore, varying ages for AD outcome assessments were used in the included studies, and we could not pool results by different age groups. The outcome of AD was determined using various measures across studies, potentially leading to bias due to the misclassification of patients with AD. Importantly, our study was limited to articles published in English, excluding abstracts, which may introduce unforeseen and immeasurable bias into our meta‐estimates.

All the included studies were adjusted for sex, age, and maternal history of allergic diseases, which are important factors associated with AD onset. However, tobacco exposure, pharmacological treatment for mental disorders, and alcohol intake have been controlled for in several studies. Although depression and anxiety are indicative of mood states, importantly, they are related to lifestyle choices, especially smoking and alcohol consumption. These behaviors are well‐recognized risk factors for the development of AD.^53^ Pharmacological treatments for mental disorders during pregnancy and their associations with allergic diseases in offspring are still disputed. A study examining the risk of asthma in offspring after antidepressant use during pregnancy found that only the use of older antidepressants was associated with an increased risk of asthma.^54^ However, these studies did not describe the treatment of individuals experiencing stress and were not considered in the meta‐analysis because of the limited number of studies. A published meta‐analysis reported that prenatal maternal psychosocial stress was associated with an increased risk of asthma in the offspring.^55^ Children having one atopic condition are at risk of having other atopic diseases. Importantly, the included studies did not specify whether patients with AD had other atopic comorbidities; this omission could obscure the true relationship that maternal stress/distress was related to other atopic comorbidities, but not AD.

## 5 CONCLUSION

This systematic review and meta‐analysis found a slightly increased risk of AD in offspring exposed to maternal stress/distress during pregnancy. Children experiencing stress/mental ill health also had a higher risk of AD. However, there is a lack of evidence on the effects of paternal, postnatal, and stress exposure in different trimesters and their association with AD. Further studies, meticulously designed, are required to confirm the relationship, enabling the application of early intervention and treatment for children at high risk of AD. Moreover, exploring the underlying mechanisms of these associations may provide a new direction for AD treatment.

## AUTHOR CONTRIBUTIONS

Conception of the work: Jichong Huang, Ting Ting Zhu Literature search: Jichong Huang, Yuan Ai, Data analysis: Jichong Huang, Yuan Ai, Ting Ting Zhu Drafting the article: Yuan Ai, Ting Ting Zhu, Critical revision of the article: Ting Ting Zhu. Final approval of the version to be published: All authors.

## CONFLICT OF INTEREST STATEMENT

The authors declare that they have no competing interests.

## CONSENT FOR PUBLICATION

Not applicable.

## Supporting information

Supporting Information S1

## Data Availability

The data used in our study were derived from publicly available data. The data sources of these data are described in the Tables and in Materials and Methods section, respectively.
