# Supplementary material for: Early exposure to maternal stress and risk for atopic dermatitis in children: A systematic review and meta‐analysis
Source: Clin Transl Allergy. 2024 Mar 15;14(3):e12346. doi: 10.1002/clt2.12346 (PMC10941798; doi:10.1002/clt2.12346)

**TITLE** Early exposure to maternal stress and risk for atopic dermatitis in child: a systematic review and meta-analysis

**Contents**

sFigure 1. Flow chart of the literature search and selection process---------------**page 2**

Principles of PICO and Search strategy-----------------------------------------------**page 3**

sTable 1. Main results of included studies ------------------------------------------**page 4-9**

sTable2. The Detailed Assessment Process of Every Included Article (score of NOS ) --------------------------------------------------------------------------------------------- **page 10**

sFigure 2. Forest plot for the association between adverse life events in early life and the development of atopic dermatitis in children----------------------------------- **page 11**

**sFigure 1. Flow chart of the literature search and selection process**

## Identification

## Eligibility

## Included

## Screening

Records identified through database searching PubMed (n = 93) Web of Science (n = 284) EMBASE (n =862) PsycINFO(n =40)

Additional records identified through other sources
(n = 16)

Records from study search
(n =1295)

Records excluded for duplicated studies
(n =235)

Records screened
(n =1060)

Records excluded for clearly irrelevant, review, case reports
(n =1020)

Full-text articles excluded with reasons (n = 18): no eczema data (13), cross-sectional study (3), adult eczema (1), overlapping populations (1)

Full-text articles assessed for eligibility
(n = 40)

Studies included in quantitative synthesis (meta-analysis)
(n = 22)

**1. PICO**

**Participants/population**: children with or without AD

**Interventions/exposures**: Any stress exposure (eg. Natural disasters, war) or adverse life events (eg. Bereavement, job strain, parental or personal conflicts, etc.) before eczema diagnosis (in the uterus including maternal or paternal exposure, after delivery)

**Comparators**: children who did not experience any stressful life event in the study period

**Types of study to be included**: Case-control, cohort

**Main outcomes**: Incidence of AD in children

**Measures of effect**: Odds ratios for odds of AD in exposed stress relative to unexposed controls.

**2. Search strategy**

**PubMed ( Limit to English)**

(( “prenatal”[Title/Abstract]) OR (“pregnancy”[Title/Abstract]) OR (“maternal”[Title/Abstract]) OR (“parents”[Title/Abstract]) OR (“paternal”[Title/Abstract]) OR (“gestation”[Title/Abstract]) OR (“postnatal”[Title/Abstract]) OR (“early life”[Title/Abstract]) OR (“fetal”[Title/Abstract]) OR (“antenatal”[Title/Abstract])

AND( “stress”[Title/Abstract]) OR (“distress”[Title/Abstract]) OR (“mental”[Title/Abstract]) OR (“mood”[Title/Abstract]) OR (“psychiatric”[Title/Abstract]) OR “psychological”[Title/Abstract]) OR (“anxiety”[Title/Abstract]) OR (“depression”[Title/Abstract]) OR (“job”[Title/Abstract]) OR (“divorce”[Title/Abstract]) OR (“adverse life”[Title/Abstract]) OR (“negtive life”[Title/Abstract])

AND ("Eczema"[MeSH Terms] OR "Eczema, Dyshidrotic "[MeSH Terms] OR" Dermatitis, Atopic "[MeSH Terms] " OR "eczema"[Title/Abstract] OR " atopic dermatitis"[Title/Abstract] OR "atopic eczema"[Title/Abstract] OR "AD"[Title/Abstract])

**Ovid PsycINFO and Web of Science (Limit to English)**

((“prenatal” OR “postnatal” OR “maternal” OR “pregnancy” OR “gestation”) AND (“stress” OR “distress” OR “anxiety” OR “depression” OR “mental” OR “mental”) AND (“eczema” OR “dermatitis” OR “atopic dermatitis”))

**Ovid EMBASE ( Limit to Human and English)**

((“prenatal” OR “postnatal” OR “maternal” OR “pregnancy” OR “gestation”) AND (“stress” OR “distress” OR “anxiety” OR “depression” OR “mental” OR “mental”) AND (“eczema” OR “dermatitis” OR “atopic dermatitis”))

| **sTable1. Main results of included studies** | | | | | | | |
| --- | --- | --- | --- | --- | --- | --- | --- |
| **Study** | **Exposure: Measures** | **Outcome: Measures** | **Effect measure/Analysis** | **Key results** | | **Methods/Variables used for confounder control** |  |
| **Prepregnancy** | | | | | | |  |
| El-Heis 2017 [20] | Maternal stress and mood; self-reported measures: GHQ-12, EPDS | eczema assessed by trained research nurses (< 2 years) | OR and 95%CI, eczema at 6 months  1.postnatal depression: 1.01 (0.99-1.03)  2. Stress in life affected health 1.10 (0.98-1.24)  3 Stress in the past 4 weeks 1.13 (1.01-1.28)  eczema at 12 months 1.postnatal depression: 1.01 (0.99-1.04)  2. Stress in life affected health 1.08 (0.96-1.23)  3 Stress in the past 4 weeks 1.12 (0.99-1.28) | | | maternal age at birth, education, smoking in pregnancy, parity and eczema, and infant sex, gestational age, season of birth and breastfeeding duration |  |
| **During pregnancy** | | | | | | |  |
| Cheng 2015 [21] | Maternal psychological state; self-reported measures: EPDS and STAI | Physician diagnosis eczema within 12 months | OR and 95%CI, 1.depression (EPDS≥15 vs <15) 1.13 (0.43–2.99) 2. anxiety (STAI≥41 vs <41) 1.01 (0.56–1.81) | | | gender, BW, GA, ethnicity, maternal age, maternal BMI, education, marital status, household monthly income, parental eczema history, maternal asthma history, maternal pre-pregnancy smoking and passive smoking during pregnancy |  |
| Shen 2020 [22] | Maternal prenatal stress; self-reported measures: 10-item Perceived Stress Scale (PSS) | Parent-reported UK Working Party diagnostic criteria standard questionnaire at 6 months | OR and 95%CI, 1.High stress vs low stress 1st trimester: 0.73 (0.50–1.07); In the 2nd trimester: 1.56 (1.08–2.25); In the 3rd trimester: 1.23(0.86–1.75); 2.Increased vs no increased from the 1st to the 2nd trimester: 2.05 (1.33–3.15) from the 2nd to the 3rd trimester: 0.99 (0.56–1.74) from the 1st to the 3rd trimester: 1.92 (1.22–3.00) | | | maternal age at delivery, ethnicity, education, family income, parity, gestational diabetes mellitus, gestational hypertension, and parental history of allergic diseases. |  |
| Shi 2023 [13] | Maternal perceived stress, anxiety, and depression; self-reported measures: LESPW, SAS, and CES–D | maternal-reported the ISAAC | OR and 95%CI 1.high stress during early pregnancy 1.30 (1.01–1.67); 2.high stress during late pregnancy 1.64 (1.14–2.36) | | | maternal age at delivery, socioeconomic status, maternal parity, exposure to second-hand smoke during pregnancy, family history of asthma, child’s birth weight, gestational age |  |
| Larsen 2014 [23] | Maternal psychosocial work environment; maternal self-reported telephone interview | parental reported questionnaire :“ever AD” (positive response to the question: “Has he/she had pruritic skin eruption?”) or itchy rash in the locations typical for AD | OR and 95%CI, (high vs low) 1.High strain during pregnancy 1.15 (1.02-1.31) 2. Active strain 1.07 (0.99-1.15) | | | maternal age, parity, body mass index, smoking, alcohol intake, gestational age at interview, furry animal ownership, maternal atopic disposition, pain killers, antibiotics, folic acid, gender, and small-for-gestational-age |  |
| Wang 2013 [9] | Maternal employment (work stress, work hours) self-reported measures | parental reported questionnaire: (Physician-diagnosed eczema ever) | OR and 95%CI, 1.Work during pregnancy 1.38 (1.25-1.53) 2. Work stress (high vs low) 1.34 (1.16-1.54) | | | sex, maternal atopy, maternal education level, maternal smoking, family income, number of siblings, and residence location. |  |
| Elbert 2017 [24] | Parental psychiatric symptoms (overall, depressive, anxiety) during pregnancy, self-reported measures: the Brief Symptom Inventory and 2 symptom scales | parental reported ISAAC | OR and 95%CI, (yes vs no) Maternal 1. Overall psychiatric symptoms 1.21 (1.05, 1.39); 2. Anxiety symptoms 1.35 (1.04, 1.76); 3. Depression 1.29 (1.02, 1.64) Paternal 1. Overall psychiatric symptoms 1.29 (0.72, 2.33) 2. Anxiety symptoms 1.11 (0.66, 1.87) 3. Depression symptoms 1.27 (0.83, 1.95). | | | maternal age and BMI at enrollment, education, ethnic origin, history of allergy, eczema or asthma,parity, pet keeping, , smoking, and child's sex, gestational age and birth weight, maternal psychiatric symptoms during pregnancy |  |
| Smejda 2018 [25] | Maternal stress during pregnancy, self-reported measures: PSS, SRRS | parental reported questionnaire and medical record | OR and 95%CI, 1.Perceived Stress Scale 0.98 (0.93–1.04) 2.Social Readjustment Rating Scale 0.99 (0.92–1.06) | | |  |  |
| Senter 2021 [36] | Maternal stress during pregnancy, self-reported measures: 14-item CDC PRAMS SLE survey | Maternal reported ISAAC | risk ratios and 95%CI, (yes vs no) 1.Current eczema: 1.08 (0.89-1.31) 2.location-specific AD 1.09 (0.78-1.52) 3. Ever AD 0.97(0.87-1.09). | | | sex, age, maternal education, household income, maternal race, maternal ethnicity, and maternal history of atopy, maternal age at delivery, maternal current stress, recruitment site, other children in home, and prenatal farm animal exposure |  |
| Wen 2011 [26] | maternal mental status during pregnancy, self-reported measures: 36 Health Survey’ (IQOLA SF-36 Taiwan standard version 1.0) | maternal reported interviews: have physician-diagnosed AD ever?) | OR and 95%CI, maternal stress (high vs low) 2.3 (1.1–5.3) | | | gender, maternal education, and parental history of atopic diseases |  |
| Hartwig 2014 [27] | maternal negative life events during pregnancy, self-reported measures: 10 typically stressful life events taken from a broader life-stress inventory | self-diagnosed or ever having been diagnosed with eczema by a physician | OR and 95%CI, Eczema at 6y 1. life events <18 weeks (≥3 vs 0) 1.41(0.61-3.29) 2. life events 18-34 weeks 2.38(0.63-2.19) Eczema at 14y 1. life events <18 weeks (≥3 vs 0) 1.18(0.54-2.60) 2. life events 18-34 weeks 4.19 (1.97-8.89) | | | sex, preterm delivery, low BW, multiple birth, parity, maternal age, prenatal tobacco smoke exposure, prenatal exposure to paint, dust and air pollution, problems during pregnancy (diabetes, steroid intake), use of antibiotics or acetaminophen-based medication in pregnancy, breastfeeding, maternal education, maternal or paternal history of eczema, postnatal tobacco smoke exposure, cat or dog ownership, postnatal maternal life events. |  |
| Chang 2016 [28] | Prenatal depression, anxiety, and distress, self-reported measures: CESD, STAI-T, K6 | 1.specialists who made a clinical diagnosis after a detailed history and physical examination 2.Korean version of the ISAAC | HR and 95%CI, 1.Prenatally depressed 1.31 (1.02-1.69); 2.Prenatally anxious 1.41 (1.06-1.89); 3.Prenatal distress 1.86 (1.06-3.26) | | | maternal age, educational level, delivery method, birth season (only in COCOA), maternal history of allergic diseases, and child’s sex |  |
| Sausenthaler 2009 [7] | Stress-related maternal factors obtained from the Mutterpass, a maternity certifi cate routinely completed in obstetrical practices. | parents reported a physician’s diagnosis of eczema | OR and 95%CI, Stress-related factors (yes vs no) 0-2y: 1.48 (0.95-2.30); 0-5y: 1.21 (0.76-1.91); 0-6y: 1.13 (0.71-1.79) | | | study center, maternal education, maternal age at delivery, and family history of atopy |  |
| Braig 2016 [29] | Maternal stress, depression, and anxiety during pregnancy; self-reported measures: SSCS-TICS, HADS, PRAQ-R | Self-administered parental questionnaires: SCORAD | OR and 95%CI, 1. SSCS-TICS(upper vs low quarter); 1.5 (1.0, 2.3) 2. HADS-A(≥8 points); 1.4 (1.0, 2.0) 3. HADS-D(≥8 points), 1.1 (0.5, 2.1) 4. PRAQ-R(upper vs low quarter) 1.5 (0.9, 2.4) | | | child sex, gestational age, maternal and paternal atopy, maternal smoking during pregnancy, maternal BMI, maternal age, child birthweight |  |
| Hamann 2019 [37] | parental psychiatric symptom during pregnancy; identified through the Civil Person Register | Data record in Danish National Health Service Register | OR and 95%CI, 1.maternal depression 1.15 (1.00-1.33) 2.maternal anxiety 1.38 (1.12-1.71) 3.maternal contacts for psychiatric care1.02 (0.99 1.05)  4.Paternal depression 1.16(1.06 1.26) 5.Paternal anxiety 1.10(0.96 1.27) 6.Paternal contacts for psychiatric care 0.99(0.95 1.03) | | | Matched factors: birthday, sex and age at diagnosis or healthcare encounter. |  |
| Puosi 2021 [30] | Maternal prenatal psychological distress, self-reported measures: EPDS and SCL-90 | doctor-diagnosed eczema according to the ISAAC | OR and 95%CI, "Consistently Low" as reference 1.EPDS (Consistently High) 0.93 (0.41–2.09) 2.EPDS (High and Decreasing) 1.52 (0.78–3.00) 3.SCL-90 (High and Decreasing) 1.76 (0.96–3.26) 4. SCL-90 (Moderate and Increasing) 1.62 (0.94–2.79) | | | child's sex, maternal history of atopic diseases, and parental education level |  |
| Letourneau 2017 [31] | Maternal psychological distress (depression, anxiety and stress) during pregnancy and postnatal, self-reported measures: EDS, SCL-90-R, PSAS and SLEQ. | Physician-diagnosed eczema was maternal reported at age 18 months | OR and 95%CI, (yes vs no) 1.Maternal unresponsiveness 1.35 (1.05–1.73) 2.Postnatal depression 0.86 (0.74–1.00) 3.Pregnancy specific anxiety 2.78 (1.04–7.39) 4.Postnatal anxiety1.16 (1.01–1.33) | | | maternal sensitivity, postnatal depression and anxiety, pregnancy specific anxiety, maternal asthma |  |
| van der Leek 2020 [14] | Prenatal and postnatal distress based on at least one health care contact or prescription of medications for depression or anxiety | more than one physician visit for AD | OR and 95%CI, maternal distress(yes vs no)  1. prenatal1.27 (1.11-1.46)  2. Postnatal, self-limiting1.01 (0.81-1.25)  3. Postnatal, recurrent1.28 (1.11-1.48)  4. Postnatal, late-onset1.19 (1.06-1.34) | | | sex, mode of delivery, low birth  weight, preterm birth, newborn respiratory distress, maternal age, first pregnancy, maternal asthma and atopy, maternal smoking during pregnancy, urban location, low household income, and infant  antibiotic use during infancy |  |
| **Postnatal** |  |  |  |  |  |  |  |
| McKenzie 2020 [32] | childhood abuse and household dysfunction at 5-,9-,15-year old; caregivers responded scale: Parent-Child Conflict Tactics Scale | Parent-reported questionnaire | OR and 95%CI, number of adverse life event at 5y ≥ 3 ACE score 2.10 (1.52–2.89) at 9y ≥ 3 ACE score 1.48 (1.09–2.01) at15y ≥ 3 ACE score 1.21 (0.84–1.75) | | | child's sex, race/ethnicity, household income, and history of asthma |  |
| Bockelbrink 2006 [33] | stressful life events; self-reported measures: Did you experience a separation or divorce from your partner? | Parent-reported questionnaire: physician-diagnosed eczema at 4 years | OR and 95%CI, stressful life events (yes vs no) 1.Death of a family member 0.67 (0.43–1.04); 2.Divorce/separation1.86 (1.09–3.19); 3.Unemployment 1.03 (0.52–2.03) | | | maternal school education, parental history of atopy, older siblings. |  |
| Wang 2016 [34] | postpartum depression and maternal mental health index; self-report questionnaire; the SF-36 | Parent-reported questionnaire: physician-diagnosed eczema | OR and 95%CI, (yes vs no) 1.Maternal stress (high vs low) 1.08 (0.92–1.28) 2. Postpartum depression 1.42 (1.21–1.66) | | | gender, birth weight, family history of atopy, maternal education, prenatal maternal stress, breastfeeding, family income, number of siblings, help for children care, residence location |  |
| Yoon 2018 [35] | Perinatal NLEs; self-report questionnaire; an NLEs score | parental reported ISAAC | OR and 95%CI, (highest vs lowest group) 1. Symptoms during the last 12 months, female 2.15 (1.24–3.71); male 1.26 (0.77–2.06) 2. Treatment during the last 12 months, female 2.08 (1.15–3.79); male 1.17 (0.67–2.05) 3.Ever asthma female 1.48 (0.78–2.80); male 0.89 (0.48–1.63) | | | maternal age, gestational age, environmental tobacco smoke exposure, parental history of allergic diseases, and maternal education level |  |
| The screening scale of the Trier Inventory of Chronic Stress (SSCS-TICS); Hospital Anxiety and Depression Scale (HADS); Pregnancy Related Anxiety Questionnaire (PRAQ-R); Scoring Atopic Dermatitis Score (SCORAD); Center for Epidemiological Studies-Depression (CESD); State-Trait Anxiety Inventory-Trait subscale (STAI-T); the Kessler Six-question Psychological Distress Scale (K6); International Study of Asthma and Allergies in Childhood (ISAAC); the Edinburgh Postnatal Depression Scale (EPDS); 12 item General Health Questionnaire (GHQ-12); birthweight (BW); body mass index (BMI); Depression Scale (EDS), Symptom Checklist-90 item-Revised (SCL-90-R); Pregnancy Specific Anxiety Scale (PSAS); Stressful Life Events Questionnaire (SLEQ); 14-item Centers for Disease Control Prevention (CDC); Pregnancy Risk Assessment Monitoring System (PRAMS); 10-item Perceived Stress Scale (PSS); The Life Events Scale for Pregnant Women (LESPW); Self-Rating Anxiety Scale (SAS); The Chinese version of the Center for Epidemiologic Studies–Depression Scale (CES–D); Social Readjustment Rating Scale(SRRS); the Taiwanese version of the short form 36 (SF-36); negative life events (NLE) | | | | | | | |

**sTable2. The Detailed Assessment Process of Every Included Article (NOS)**

| study | Selection 1) | Selection 2) | Selection 3) | Selection 4) | Comparability 1) | Comparability 2) | Outcome 1) | Outcome 2) | Outcome 3) | Score |
| --- | --- | --- | --- | --- | --- | --- | --- | --- | --- | --- |
| Cohort |  |  |  |  |  |  |  |  |  |  |
| Bockelbrink 2006 [33] | 1 | 1 | 1 | 1 | 1 | 0 | 0 | 1 | 1 | 7 |
| Braig 2016 [29] | 1 | 1 | 1 | 1 | 1 | 1 | 1 | 1 | 1 | 9 |
| Chang 2016 [28] | 1 | 1 | 1 | 1 | 1 | 1 | 1 | 1 | 0 | 8 |
| Cheng 2015 [21] | 1 | 1 | 1 | 1 | 1 | 1 | 1 | 1 | 0 | 8 |
| Elbert 2017 [24] | 1 | 1 | 1 | 1 | 1 | 1 | 1 | 1 | 1 | 9 |
| El-Heis 2017[20] | 1 | 1 | 1 | 1 | 1 | 1 | 1 | 1 | 1 | 9 |
| Hartwig 2014 [27] | 1 | 1 | 1 | 1 | 1 | 1 | 0 | 1 | 0 | 7 |
| Larsen 2014 [23] | 1 | 1 | 1 | 1 | 1 | 1 | 0 | 1 | 1 | 8 |
| Letourneau 2017 [31] | 1 | 1 | 1 | 1 | 1 | 0 | 1 | 1 | 1 | 8 |
| McKenzie 2020 [32] | 1 | 1 | 1 | 1 | 1 | 0 | 0 | 1 | 0 | 6 |
| Puosi 2021 [30] | 1 | 1 | 1 | 1 | 1 | 0 | 1 | 1 | 1 | 8 |
| Sausenthaler 2009 [7] | 1 | 1 | 1 | 1 | 1 | 0 | 1 | 1 | 1 | 8 |
| Senter 2021 [36] | 1 | 1 | 1 | 1 | 1 | 1 | 1 | 1 | 1 | 9 |
| Shen 2020 [22] | 1 | 1 | 1 | 1 | 0 | 1 | 1 | 0 | 1 | 7 |
| Shi 2023 [13] | 1 | 1 | 1 | 1 | 1 | 1 | 1 | 1 | 1 | 9 |
| Smejda 2018 [25] | 1 | 1 | 1 | 1 | 1 | 0 | 1 | 1 | 0 | 7 |
| van der Leek 2020 [14] | 1 | 1 | 1 | 1 | 1 | 1 | 1 | 1 | 1 | 9 |
| Wang 2013 [9] | 1 | 1 | 1 | 1 | 1 | 0 | 1 | 1 | 1 | 8 |
| Wang 2016 [34] | 1 | 1 | 1 | 1 | 1 | 0 | 1 | 0 | 1 | 7 |
| Wen 2011 [26] | 1 | 1 | 1 | 1 | 1 | 0 | 1 | 1 | 1 | 8 |
| Yoon 2018 [35] | 1 | 1 | 1 | 1 | 1 | 1 | 1 | 1 | 0 | 8 |
| Case-control |  |  |  |  |  |  |  |  |  |  |
|  | Selection 1) | Selection 2) | Selection 3) | Selection 4) | Comparability 1) | Comparability 2) | Exposure 1) | Exposure 2) | Exposure 3) | Score |
| Hamann 2019 [37] | 1 | 1 | 1 | 1 | 1 | 0 | 1 | 1 | 0 | 7 |

**sFigure 2. Forest plot for the association between adverse life events in early life and the development of atopic dermatitis in children.**


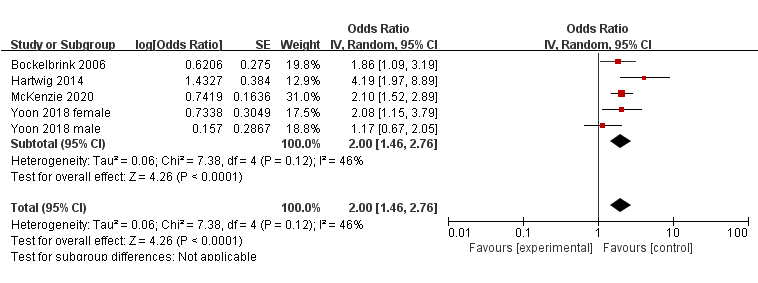

Supplement: Supplementary file 1 — Supporting Information S1 [file CLT2-14-e12346-s001.docx]
